# Opening new perspectives on microbiology

**DOI:** 10.1002/mbo3.1

**Published:** 2012-03

**Authors:** Pierre Cornelis


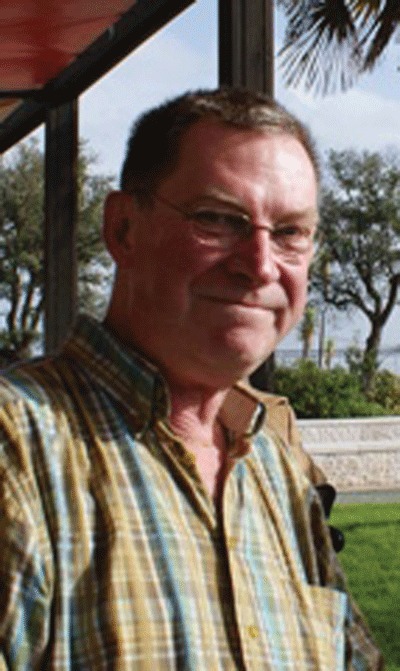


I am pleased and honored to introduce MicrobiologyOpen, a new journal from Wiley-Blackwell. Wiley-Blackwell already publishes an impressive portfolio of microbiology journals, a number of them for leading societies, including some of the most read and most cited journals serving the discipline. These journals are highly regarded among microbiologists. Why then a new journal? The term “open” has two meanings: first, MicrobiologyOpen is an open access journal, but, more importantly, it also means that it is open to all aspects of microbiology. The leading journals in the Wiley-Blackwell microbiology portfolio target specific microbiology audiences; MicrobiologyOpen launches specifically to serve the broad microbiology community.

Microbiology has changed dramatically during the last 10 years and has undergone very exciting developments in this postgenomic era. The massive quantity of genomic and metagenomic information that is now available raises many questions and opens new perspectives. We should not forget that many genes code for proteins of unknown function and it is a challenge for scientists in the field to determine what these proteins do by combining different approaches, including genetics, biochemistry, physiology, proteomics, metabolomics, to cite only a few. MicrobiologyOpen is a response to the growth of research being undertaken and the data now available. It is clear that the community will benefit from the increased flexibility provided by the online only, open access format, since neither the authors’ voices nor the journal's development will be curtailed by length restrictions or page budgets.

Manuscripts dealing with all types of microorganisms (Archaea, Bacteria, Eukarya) will be welcome, and virology is certainly not forgotten, since the journal will publish articles dealing with bacterial and archaeal phages as well as papers on viruses infecting eukaryotic microorganisms. Being a consciously interdisciplinary journal, MicrobiologyOpen encourages and will consider equally papers dealing with fundamental aspects and with applied research.

The journal welcomes direct submissions that further our understanding of microbial interactions and microbial processes. We have an editorial board of excellent scientists with different and complementary expertise, and we are conscious of the need to maintain a high scientific standard. Together we aim at reaching a level of excellence for MicrobiologyOpen, which means that only manuscripts of high scientific interest and value will be accepted. All submissions will be subject to stringent but swift peer review by at least two referees chosen among the members of the Editorial Board and/or by independent external experts. MicrobiologyOpen also collaborates with other journals published by Wiley-Blackwell, including society-owned journals, in order to offer authors of high-quality work that these leading journals are unable to publish an opportunity to transfer their manuscripts along with the original reviews for consideration by MicrobiologyOpen.

We aim at a rigorous, fair, and rapid processing of the manuscripts submitted to MicrobiologyOpen, which means that the authors will get a decision within a few weeks of submission. In addition, the journal will utilize an XML editing tool to provide fast turnaround to proofs and handle author corrections quickly, with the result that the time between acceptance and publication will also be considerably shortened.

I would like to thank the scientists who accepted with enthusiasm to become members of the editorial board of the journal. To become the first Editor-in-Chief of this new journal is certainly an enormous challenge for me and equally for the members of the Editorial Board. We will together do our best to make MicrobiologyOpen one of the most respected journals in microbiology.

